# Leaf and Root Functional Traits of Woody and Herbaceous Halophytes and Their Adaptations in the Yellow River Delta

**DOI:** 10.3390/plants14020159

**Published:** 2025-01-08

**Authors:** Yan Wang, Hao Wu, Jian Wang, Liqiang Mu, Zhongyue Li

**Affiliations:** 1 State Forestry and Grassland Administration Key Laboratory of Silviculture in Downstream Areas of the Yellow River, College of Forestry, Shandong Agricultural University, Tai’an 271000, China; wangyan_nefu@126.com; 2 School of Forestry, Northeast Forestry University, Harbin 150040, China; 3 Chaoyang Ecological Environment Affairs Service Center, Chaoyang 122000, China

**Keywords:** Yellow River Delta, halophytes, leaf and root, functional trait, morphology, anatomy

## Abstract

Leaves and roots perform assimilation, supporting plant growth and functionality. The variations in their functional traits reflect adaptive responses to environmental conditions, yet limited information is available regarding these trait variations and their coordination in saline environments. In this study, 18 common woody and herbaceous halophyte species from the Yellow River Delta were collected, and their leaf and root functional traits were assessed and compared. Our results showed that, compared with herbaceous species, woody species had greater root diameter, cortex thickness, and stele diameter, but lower specific root length and leaf area. Meanwhile, root diameter was strongly correlated with cortex thickness and stele diameter; leaf thickness was also tightly related with palisade tissue thickness. However, fewer correlations were found between paired leaf and root traits in either herbaceous or woody species, indicating that the variations in leaves and roots appeared relatively independent, which might be related to the different abiotic environmental conditions experienced by above- and belowground organs. These results highlight that woody species tended to be more conservative in resource acquisition and establishment; meanwhile, the herbaceous ones were acquisitive. Such patterns show the contrasting survival strategies of different plant taxa, which also provide valuable insights for future vegetation restoration efforts in this salinized region.

## 1. Introduction

Nowadays, the expansion of saline-alkali land has severely impacted plant growth and destabilized agricultural and forest ecosystems globally [1,2]. In salinized habitats, the specialization of leaves or/and roots facilitates their adaptation to stressful conditions. Based on ecological, physiological, and eco-physiological criteria, such plants that live in or have adapted to salty environments are defined as halophytes [3]. In recent decades, trait-based approaches have been widely used in ecological and evolutionary research. Meanwhile, the variations in and coordination of plant functional traits directly influence fitness through their effects on growth, reproduction, and survival, as well as ecosystem functioning [4,5,6]. Therefore, exploring the variations in root and leaf functional traits in salinized conditions with low water availability could provide crucial information for vegetation restoration and conservation in saline-alkali areas.

Generally, salinity and nutrient content are the most significant edaphic factors influencing plant growth, development, and distribution. For example, in a potted experiment, total leaf area per unit plant biomass under saline conditions was higher than that under control, while root length per unit plant mass was lower [7]. In the foredune of Brazil, the woody community exhibited a conservation strategy with low specific leaf area (SLA), thick cuticles, and high frequencies of phenolic compounds and crystals; meanwhile, herbaceous plants exhibited either acquisitive characteristics (e.g., high SLA values) or a conservative strategy (e.g., rhizome and xylopodium with starch or inulin promoting plant regrowth during favorable periods) [8]. At the same time, in the coastal regimes of the Caribbean, comparing three mangroves (*Avicennia germinans*, *Laguncularia racemosa,* and *Rhizophora mangle*) in oligohaline conditions and in euhaline conditions, i.e., relatively high salinity and low nitrogen concentrations, the same mangroves decreased their stomatal density and conductance, but increased their stomatal size [9]. Along the north bank of the Aqikesu River in China, with increased salt concentration, the leaf epidermis thickness and the palisade-to-spongy tissue ratio decreased in the dominant species, *Nitraria sibirica* and *Alhagi sparsifolia*; additionally, the looseness of the palisade tissue increased [10]. Although all the functional traits mentioned above exhibited significant intra- and interspecific variation in response to natural or artificial conditions, most investigations sampled either the leaf or the root of herbaceous or woody species; accordingly, the integration and coordination between roots and leaves in different functional plant groups is still an open issue.

The Yellow River Delta wetland is a nature reserve with important ecological functions and service values, which are significant for regional ecological protection and species diversity maintenance in the coastal area [11]. However, a coastal area is a transition zone between land and sea; anthropogenic manipulations of the hydrologic cycle and the worldwide sea level rise induce the frequent intrusion of high-salinity seawater into inlands, leading to salt accumulation in the soil [12]. The salinization of water and soil is an extremely prominent environmental problem in the Yellow River Delta, substantially influencing plant performance [13]. For instance, in the coastal-inland regions of the Yellow River Delta, the specific leaf area and leaf thickness of *Phragmites australis* decreased significantly with increased soil salinity but showed a smaller response to soil water content [14]. The lower daily flooding level induced by the high frequency of tidal inundation increased the soil salinity of marshes in the Yellow River Delta, which decreased the sexual reproduction of seedling height and the seedling density of clonal ramets in *Spartina alterniflora* [15]. However, the way in which different plant communities respond to soil salination in the Delta remains unclear.

In this study, common and widespread species were collected in the Yellow River Delta, including nine woody and nine herbaceous species. Specifically, root tips and leaves were sampled for trait analysis, and 16 morphological and anatomical traits were measured, including root diameter, specific root length, root cortex thickness, leaf thickness specific leaf area, and palisade thickness. Additionally, the variations in functional traits between different plant groups and their potential coordination were also analyzed. Our objectives were as follows: (1) to compare the functional trait variations in root tips and leaves between different plant groups; and (2) to explore the correlation between leaf and root functional traits within and/or between plant groups.

## 2. Results

### 2.1. Morphology Trait Variation

**Figure 1:**
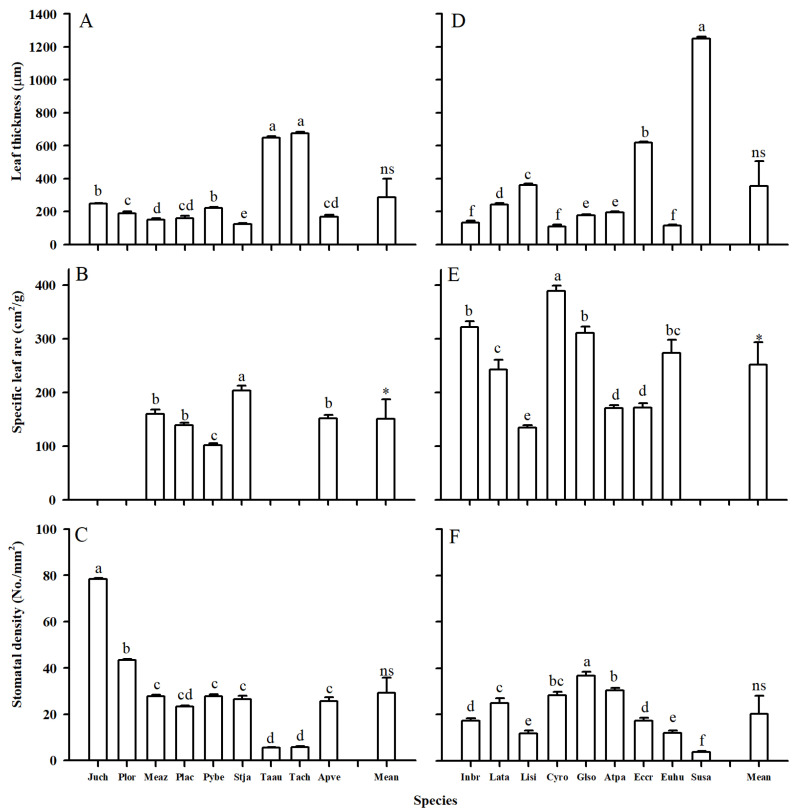
Leaf thickness, specific leaf area, and stomatal density of woody (**A**–**C**) and herbaceous species (**D**–**F**) in Yellow River Delta. The bars and error bars represent mean ± SEM. The mean represents the averaged value across the examined species within each plant group. Species abbreviations are provided in Table 2. Different lower case letters within the clusters of bars indicate significant differences (*p* < 0.05) among different species within the woody or herbaceous groups according to Fisher’s LSD test. * and ns represent *p* values less than 0.05 and larger than 0.05 for the comparison of means between woody and herbaceous species according to the *t*-test, respectively.

**Figure 2:**
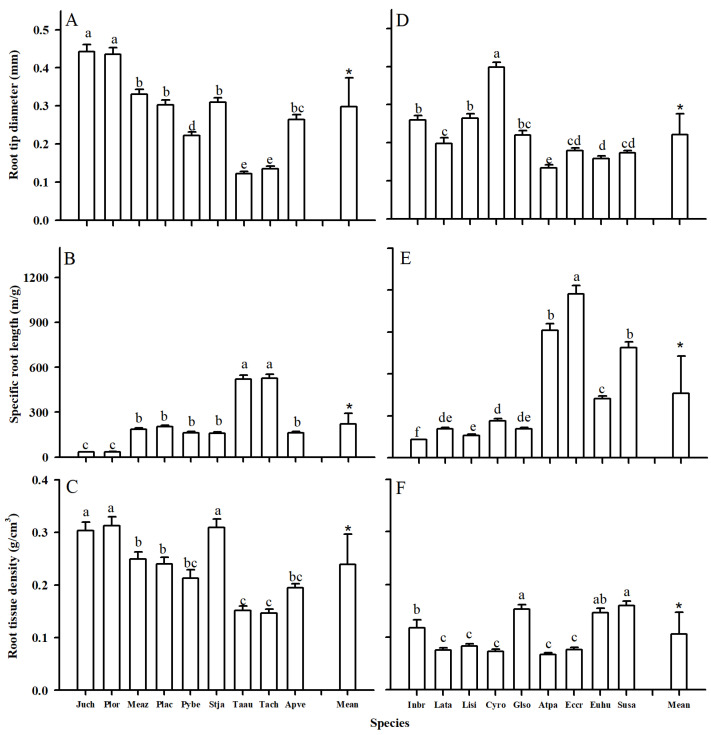
Root diameter, specific root length, and tissue density of woody (**A**–**C**) and herbaceous (**D**–**F**) species in Yellow River Delta. The bars and error bars represent mean ± SEM. The mean represents the averaged value across examined species within each plant group. Species abbreviations are provided in Table 2. Different lower case letters within the clusters of bars indicate significant differences (*p* < 0.05) among different species within the woody or herbaceous groups according to Fisher’s LSD test. * represents a *p* value less than 0.05 for the comparison of means between woody and herbaceous species according to the *t*-test.

Both leaf and root tip morphological traits showed considerable variations between two plant groups (Figure 1 and Figure 2; Table 1). Compared with woody species, herbaceous species generally had lower leaf thickness (LT), root diameter (RD), and root tissue density (RTD), but higher specific leaf area (SLA) and specific root length (SRL) (Table 1, *p* < 0.05). Generally, the morphological traits of herbaceous species also showed wider variations than woody ones, with higher values of variable coefficients (CVs) (except the SRL). For example, the LTs of woody and herbaceous species were 286.99 and 372.55 μm, with CVs of 0.75 and 1.05, respectively (Table 1).

### 2.2. Anatomy Trait Variations

**Figure 3:**
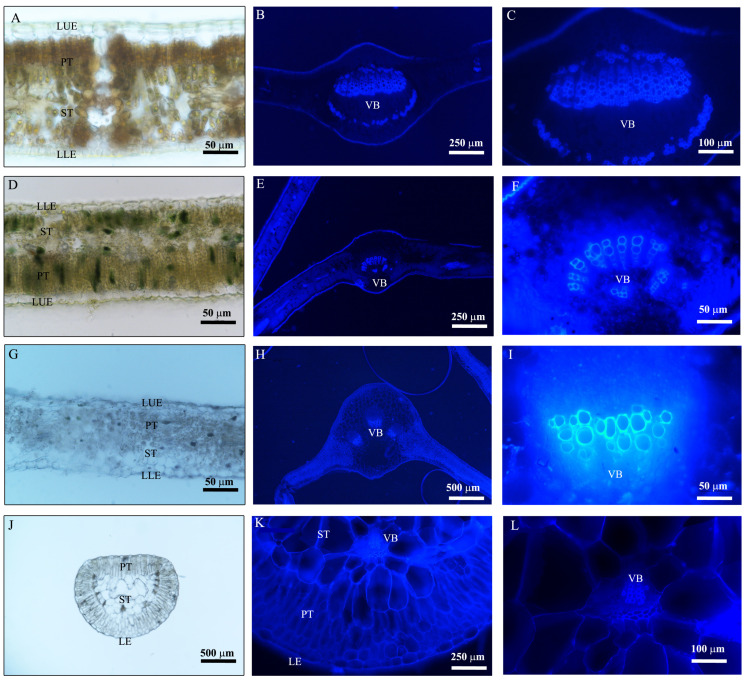
Anatomical structures of leaves in woody ((**A**–**C**), *Pyrus betulifolia*; (**D**–**F**), *Melia azedarach*) and herbaceous ((**G**–**I**), *Lactuca tatarica*; (**J**–**L**), *Suaeda salsa*) species, respectively. The images (**A**,**D**,**G**), and (**J**) were obtained under white light, and the other images under UV–blue light, respectively. LUE, leaf upper epidermis; LLE, leaf lower epidermis; LE, leaf epidermis; PT, palisade tissue; ST, spongy tissue; VB, vascular bundle.

**Figure 4:**
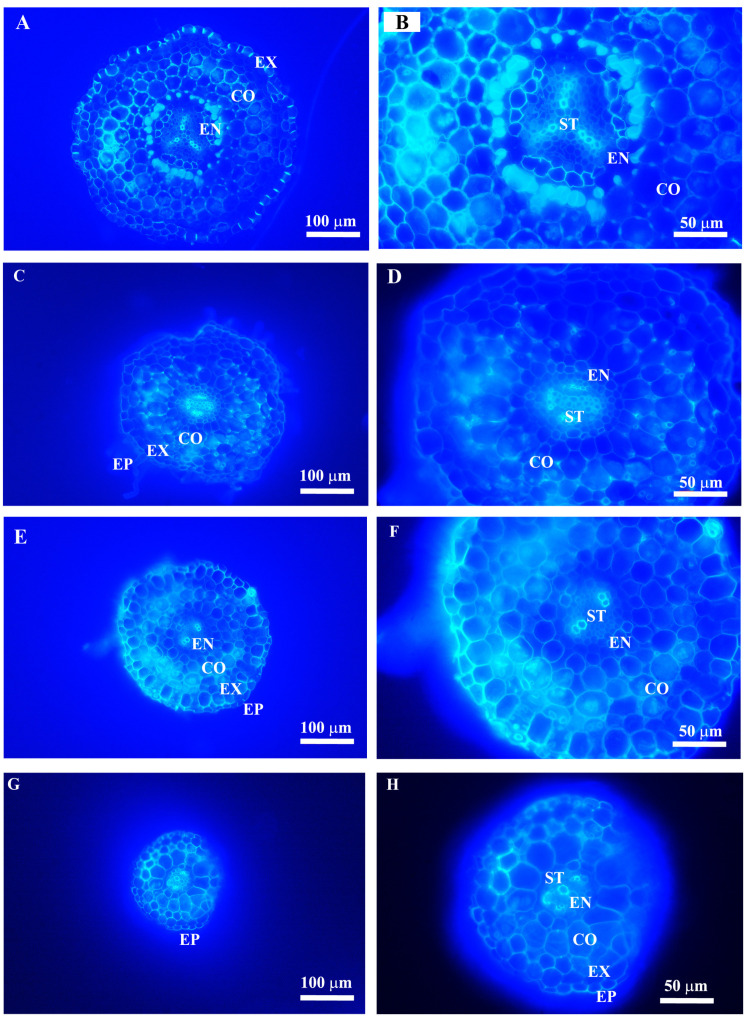
Anatomical structures of root tips in woody (**A**,**B**, *Platycladus orientalis*; **C**,**D**, *Melia azedarach*) and herbaceous (**E**,**F**, *Inula britannica*; **G**,**H**, *Euphorbia humifusa*) species, respectively. EP, epidermis; EX, exodermis; CO, cortical parenchyma cell; EN, endodermis; ST, stele.

**Figure 5:**
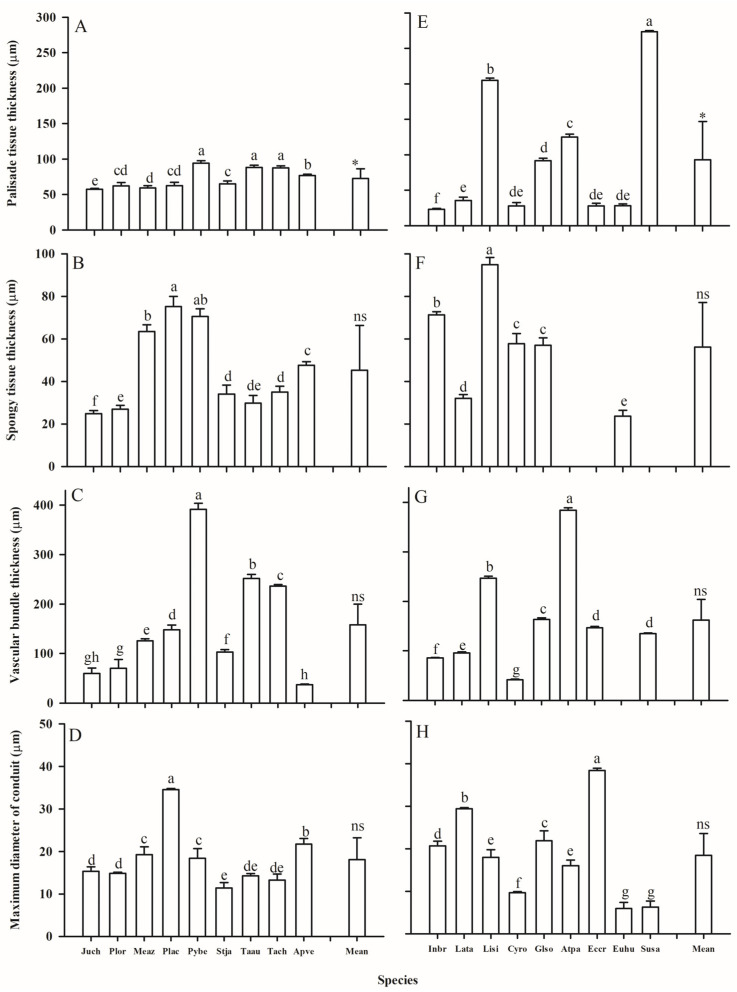
Leaf palisade and spongy thickness, vascular bundle thickness, and maximum diameter of conduits of woody (**A**–**D**) and herbaceous (**E**–**H**) species in the Yellow River Delta. The bars and error bars represent mean ± SEM. The mean represents the averaged value across the examined species within each plant group. Species abbreviations are provided in Table 2. Different lower case letters within the clusters of bars indicate significant differences (*p* < 0.05) among different species within the woody or herbaceous groups according to Fisher’s LSD test. * and ns represent *p* values less than 0.05 and larger than 0.05 for the comparison of means between woody and herbaceous species according to the *t*-test, respectively.

**Figure 6:**
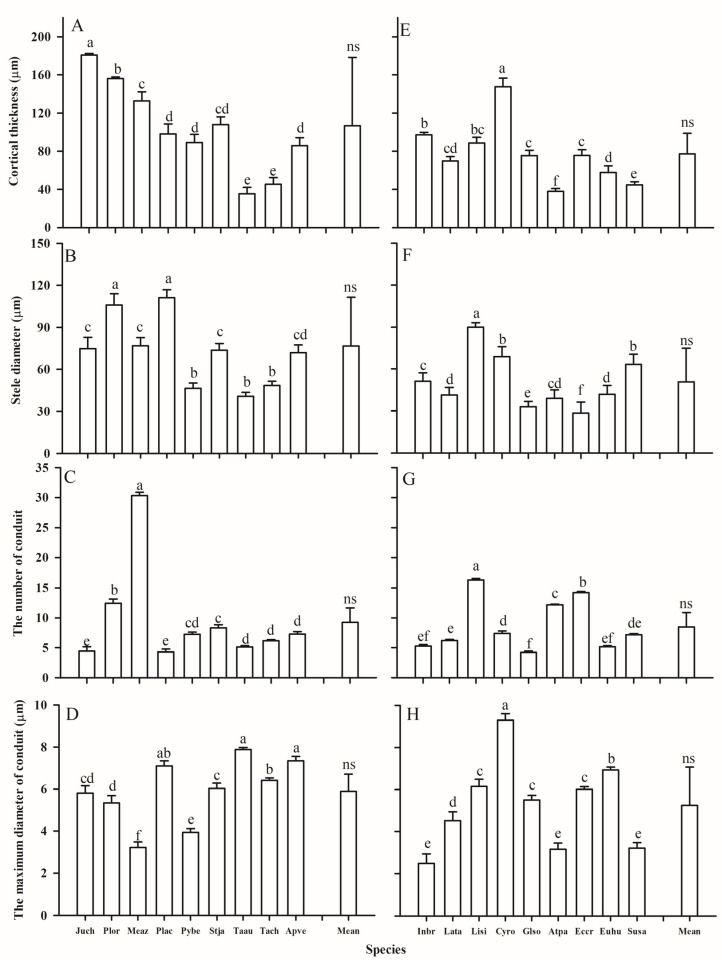
Root cortical thickness, stele diameter, and number and maximum diameter of conduits of woody (**A**–**D**) and herbaceous (**E**–**H**) species in the Yellow River Delta. The bars and error bars represent mean ± SEM. The mean represents the averaged value across the examined species within each plant group. Species abbreviations are provided in Table 2. Different lower case letters within the clusters of bars indicate significant differences (*p* < 0.05) among different species within the woody or herbaceous groups according to Fisher’s LSD test. ns represents a *p* value larger than 0.05 for the comparison of means between woody and herbaceous species according to the *t*-test.

The leaf and root anatomical traits of woody and herbaceous species showed substantial variability (Figure 3, Figure 4, Figure 5 and Figure 6, Table 1). Due to the fleshy, cylindrical leaves of *Suaeda salsa* (Figure 3C), the mean leaf palisade thickness (LPT), and spongy thickness (LST) in herbaceous species were thicker than in woody species, with CVs of 0.98 vs. 0.20, 0.46 vs. 0.44, respectively (Figure 5, Table 1). Additionally, woody species generally had slightly more (*p* = 0.302) and larger (*p* = 0.139) stomata, but significantly longer veins (*p* = 0.001) than herbaceous species. Similarly, woody species had significantly larger root cortex thickness (RCT) and root stele diameter (RSD) compared to herbaceous species (Figure 5 and Table 1, *p* ≤ 0.05).

The vascular tissue also exhibited wide variations between the two plant groups, although a significant difference was only found in the leaf vascular bundle thickness (LVT). Generally, compared with herbaceous species, woody species had wider and more conduits in the roots and thicker vascular bundles, but smaller conduits in the leaves (Table 3). Furthermore, the maximum conduit diameter in the roots and leaves of herbaceous species showed higher CVs than those of woody species (0.46 vs. 0.26, 0.58 vs. 0.38, respectively).

### 2.3. Trait Associations Within/Between Above- and Below-Ground Organs

**Figure 7:**
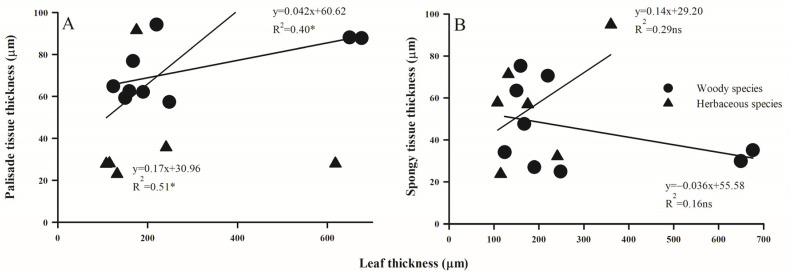
The relationship of palisade (**A**) and spongy (**B**) thickness and leaf thickness across woody and herbaceous species in the Yellow River Delta. For the significance of the correlation, ns, *p* > 0.05; *, p < 0.05, respectively.

**Figure 8:**
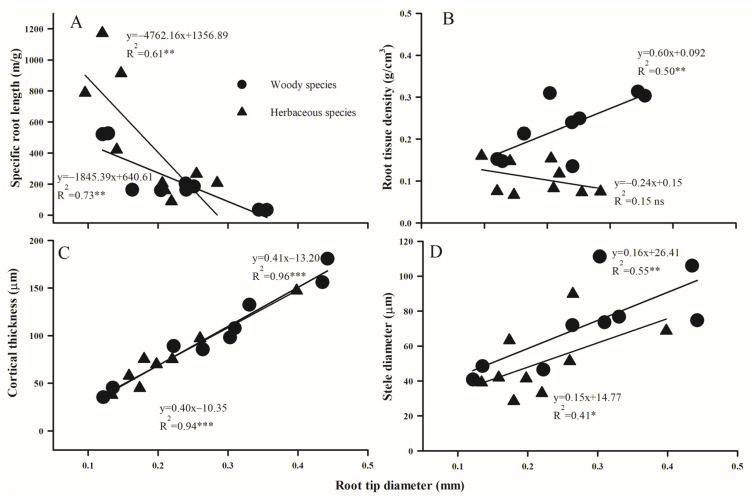
The relationship between the morphology (**A**,**B**) and anatomy (**C**,**D**) traits of roots and leaves across woody and herbaceous species in the Yellow River Delta. For the significance of the correlation, ns, *p* > 0.05; *, *p* < 0.05; **, *p* < 0.01; ***, *p* < 0.001, respectively.

**Figure 9:**
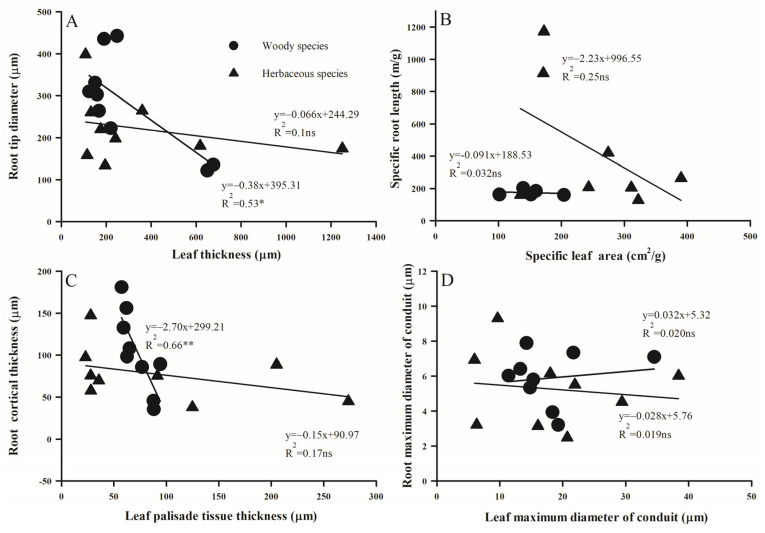
The relationship between specific root length (**A**), tissue density (**B**), cortical thickness (**C**), and stele diameter (**D**) and root diameter across woody and herbaceous species in the Yellow River Delta. For the significance of the correlation, ns, *p* > 0.05; *, *p* < 0.05; **, *p* < 0.01, respectively.

The relationship between different morphological and anatomical traits of leaves and absorptive roots exhibited substantial variation between woody and herbaceous species (Figure 7, Figure 8 and Figure 9). LPT, rather than LST, was positively related with LT in both woody and herbaceous species (Figure 7). Additionally, as RD thickened, SRL decreased significantly (Figure 8A, *p* < 0.05), and RTD increased in woody species (Figure 8B, *p* < 0.05), but decreased in herbaceous species (Figure 8B, *p* = 0.15). Anatomically, both RCT and RSD increased linearly with RD, with the slope for RCT much steeper than that for RSD (R^2^ values were 0.96 and 0.94 vs. 0.55 and 0.41 for woody and herbaceous species, respectively; Figure 8). Across above- and belowground organs, negative relationships were observed between RD and LT (Figure 9A), SRL and SLA (Figure 9B), and RCT and LPT (Figure 9C) in both plant groups, but were only significant for woody species. No obvious relationship was observed between the maximum conduit diameters in leaves and roots (Figure 9D).

**Figure 10:**
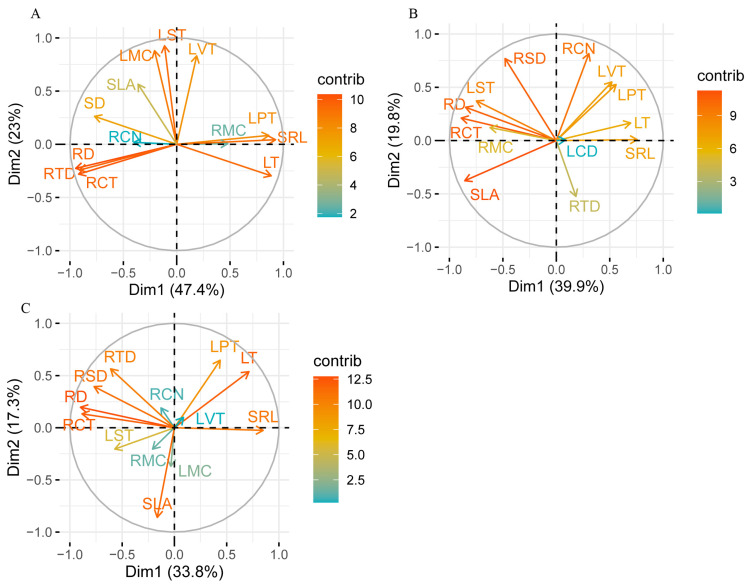
Principal component analysis of root and leaf functional traits across woody (**A**), herbaceous (**B**), and all examined (**C**) species in Yellow River Delta. RD, root tip diameter; SRL, specific root length; RTD, root tissue density; RCT, root cortical thickness; RSD, root stele diameter; RCN, number of root conduits; RMC, maximum diameter of root conduit; LT, leaf thickness; SLA, specific leaf area; LPT, leaf palisade tissue thickness; LST, leaf spongy tissue thickness; LVT; leaf vascular bundle thickness; LMC, maximum diameter of leaf conduit.

Furthermore, the results of our principal component analysis (PCA) showed that the first two trait axes explained 47.4% and 23.0% of the total variation in woody species (Figure 10A), 39.9% and 19.8% in herbaceous species (Figure 10B), and 33.8% and 17.3% in all examined species (Figure 10C), respectively. Specifically, all measured functional traits of root tips and leaves exhibited varying degrees of variation across different plant groups. For instance, in woody species, the first principal component was dominated by root traits, including SRL and RTD (Figure 10A). The second principal component, orthogonal to the first one, was dominated by leaf traits, such as LST and SLA. In herbaceous species, trait correlations were more complex; one resource-acquisition-related dimension aligned with the first component, clustering with SRL and SLA, while the resource-conservative-related dimension aligned with the second one, clustering with RTD, RSD, and the number of root conduits (RCNs). In all examined species, trait coordination resembled that of woody species to some extent, with the root and leaf traits remaining relatively independent (Figure 10C).

## 3. Discussion

### 3.1. Leaf and Root Trait Variations Between Two Groups

SLA is a key trait indicating the cost-benefit balance of leaf construction and reflects plant adaptation strategies. In this study, woody species generally exhibited lower SLA than herbaceous species, potentially due to the differences in light availability. Previous studies have demonstrated that under shaded conditions, as light intensity decreases, leaf area is maximized per unit of carbon investment to enhance light capture. This phenomenon has been widely observed across various plant life forms, including herbaceous [15,16], shrub [17,18], and woody [19,20] species. The tall trees and shrubs examined in this study could access light more readily than herbaceous species under the forest canopy, resulting in relatively higher SLA values. Additionally, under full light conditions, herbaceous species, such as *Lactuca tatarica* and *Limonium sinense*, exhibit thick leaves and low SLA; however, their dense palisade tissue enhances photosynthesis.

Typical halophytes in the Yellow River Delta, such as the woody *Tamarix chinensis* and *T. austromongolica*, and the herbaceous *S. salsa*, have distinctive trait specializations differing from other species. For instance, most *Tamarix* are stress-tolerant, exhibiting ecological adaptability to drought and salinization [21], with highly specialized scale-like leaves and lower SLA. Therefore, thicker leaves increase dry weight, reduce plasma membrane permeability under high salt conditions, and enhance salinity tolerance [21,22]. Additionally, *S. salsa* occurs in saline areas as a pioneer species, with succulent leaves functioning as adaptive organs for salt accumulation. This adaptation enables the mesophyll cells to retain sufficient water, dissolve salt ions, and maintain osmotic balance [23,24], thereby conferring high tolerance to soil salinization [23].

In this study, absorptive root tips vary widely in morphology and anatomy among different plant taxa. Compared to herbaceous species, woody ones generally have thicker root diameter, cortexes, and steles; more and wider conduits; and greater tissue density, but lower SRL. Similar patterns have been observed in previous studies. For example, a study involving 8 monocot herbaceous species, 51 eudicot herbaceous species, and 23 eudicot woody species conducted in Northeast China reported that the average root tip diameters for these three groups were 160.57, 176.87, and 193.49 µm; the cortex thicknesses were 61.83, 63.65, and 67.52 µm; and the stele diameters were 29.49, 34.97, and 40.23 µm, respectively [25]. Similar findings were also observed in potted experiments on woody and herbaceous plants [26]. Overall, this pattern aligns with the resource-acquisition and survival strategies of different plant taxa. Thicker roots with greater tissue density [27] correlate with an increased lifespan [28,29], reducing construction costs [30], which has been confirmed across diverse plant taxa such as ferns, herbaceous plants, shrubs, and trees. Therefore, as perennial species, trait syndromes in root morphology, anatomy, physiology, and lifespan are crucial for balancing investment and return over the long term [31].

Compared with these results mentioned above [25], both the examined woody and herbaceous species seemed to be thicker in root diameter in the Yellow River Delta (Table 1); such patterns seem to be general across terrestrial ecosystems, even under salinized conditions. Additionally, thicker cortexes and diameters might be related to enhanced root tolerance to salinity, which were also reported in both field observations [32] and a potted experiment [33] under saline conditions. However, it was also worth noting that in the typical halophytes, *T. chinensis* and *T. austromongolica*, as well as *S. salsa*, their thinner cortex and root diameter with high SRL may increase their resource uptake ability in salinized soil, reflecting root anatomical adaptations to salinity across different plant groups. Therefore, thicker leaves (discussed above) coupled with thinner roots would facilitate resource acquisition in a salinized soil habitat.

### 3.2. Trait Correlations

We also found that root tips with greater diameter typically had thicker cortex and stele tissues, and furthermore, variations in cortex thickness explained a larger proportion of root diameter variance compared to stele thickness. Similar structural patterns were previously observed in subtropical [34] and temperate [35] woody species, which might be general across different ecosystems, even under salinized soil conditions. There are several reasons for this. Firstly, the cortex accounts for a greater proportion of the cross-sectional area of root tips, as found by this and other studies [34,35]. Secondly, interspecific wide variations in cortex thickness were associated with evolutionary adaptation to long-term environmental changes [35,36]. In geological time, during species radiation, plants were exposed to different drastic changes, including temperature and atmospheric carbon dioxide concentrations, as well as soil nutrients [37]. Consequently, the outer cortex suffering the environmental changes directly varied widely, while the inner stele surrounded by cortex was isolated from environmental stresses, showing less variations [35], manifesting as a large cortex space conferred by thick roots in this study. Thirdly, the tightness of linkages between cortex and stele and root also reflected the trade-off between absorption and transportation. Previous studies reported that with an increase in root order, the relationship became looser for cortex thickness and root diameter, but tighter for stele and root diameter [34]. Especially for high-order secondary roots, collapsed or even disappeared cortexes decreased uptake ability, while the secondary xylem occupied the majority of the cross-sectional area and increased transportation capacity [38,39,40,41,42]. Therefore, in the root tips, as the primary absorptive roots, the different variations in root diameter contributed by the cortex and stele also reflect a functional trade-off between absorption and transport.

Additionally, the relationships in morphology and anatomy between leaf and root traits differed significantly in the two plant groups, showing only minor deviations from previous studies. For instance, a positive correlation between SLA and SRL was observed in potted seedlings of woody plants, but not in herbaceous species [27]. Similar patterns have been reported in other studies examining both woody and herbaceous species [43,44,45]; in contrast to our results, SLA and SRL showed less associations with each other in both plant groups. The uncoupled relationship might be related to the different environmental conditions experienced by leaves and roots. For example, the typical halophyte, there are scale leaves in *T. austromongolica* and *T. chinensis*, and fleshy and cylindrical leaves in *S. salsa*, which means that the lower leaf expanding area per caron investment enhances the water and sugar storage capacity for high salt tolerance. However, these species had greater SRL, i.e., longer roots under salinization conditions for resource absorption. Correspondingly, different environmental conditions experienced by leaves and roots, i.e., full light and salinized soil, respectively, possibly drive the diverse trait variation patterns. In addition, the divergence was also further reflected in the observed negative relationship between palisade and cortex thickness (although not significant in herbaceous species) in this study. Therefore, in the Yellow River Delta, unlike the trait coordination observed in previous studies [26,27,35,36,37,38,39,40,41,42,43], the reduced association between above- and belowground organs reflected that the specific modifications in leaves or/and roots for resource acquisition and environmental adaption are beneficial for plant survival and growth under stressful conditions.

## 4. Materials and Methods

### 4.1. Study Site

The study was conducted in the Yellow River Delta (117°31′–119°18′ E, 36°55′–38°16′ N), with a total area of about 26,500 Km^2^ and an altitude of 0–13 m. This site has a warm temperate sub-humid continental monsoon climate, with an average annual precipitation of 580 mm and a mean annual temperature ranging from 11.7 °C to 12.8 °C. The modern Yellow River Delta formed in 1855 with frequent changes in river channels. The soils are mainly coastal saline and tidal soils, with severe salt erosion; the salt content ranges from 0.1% to 1%. This site is the most complete and youngest wetland ecosystem in the warm temperate zone of China, containing many halophytes, which presents an advantage for examining functional trait variations and adaptations.

### 4.2. Sample Collection

In this study, nine woody and nine herbaceous species (Table 2) were collected in 2023, covering 14 families; all these species are widespread in the Yellow River Delta. Detailed information about the taxonomic list, life forms, and vegetation type for every species is supplied in Table 2; the species names were provided based on the criteria of POWO (Plants of the World Online, https://powo.science.kew.org/ (accessed on 24 December 2024)) and the traditional FOC (Flora of China); when names were in conflict, International Plant Names Index conventions were followed (IPNI; http://www.ipni.org/ (accessed on 24 December 2024)). Based on the field investigation, although the plant species varied widely in leaf morphological parameters, the ideal materials, branched root systems, provided the possibility of explore plant adaptations to salinized soil conditions. Four to five healthy individual plants were selected; for each one, leaves that were complete and free of insects were collected. Correspondingly, root branches were also sampled. Once collected, all the samples were divided into two subsamples: for the leaves, the fresh samples were scanned to determine their area, and then frozen for later drying treatment. For the roots, one subsample was immediately put on ice and transported to the laboratory within 4 h, then frozen for subsequent morphological analysis. The other parts were gently washed in deionized water (roots) or brushed to remove residual dirt (leaves), then immediately fixed in a formalin-aceto-alcohol (FAA) solution (90 mL of 50% ethanol, 5 mL of 100% glacial acetic acid, and 5 mL of 37% methanal).

### 4.3. Trait Measurement

For the morphological measurements, five scanned and frozen leaf subsamples were dried at 60 °C in the laboratory for weight measurement. Then, the specific leaf area (SLA) was calculated as the total leaf area divided by the corresponding dry mass. Due to the difficulty of measuring the leaf area of a scale leaf precisely, the SLA and vein density (VD) of *Juniperus chinensis* (Juch), *Platycladus orientalis* (Plor), *T. austromongolica* (Taau), and *T. chinensis* (Tach) were neglected, which was also the same for fleshy and cylindrical leaves in *S. salsa*. For the roots, only the unbranched root tips were collected for trait analysis, because they are primarily responsible for water and nutrient uptake [33], and are more sensitive to environmental changes [37,46]. The specific abbreviations and descriptions of all measured traits are shown in Table 2.

Four root tip subsamples of each species were scanned with an EPSON EXPRESSION 850PRO colored scanner (dots per inch = 400). The mean root diameter, total length, and volume for each species were determined with the root system analyzer software (WinRhizo 2004b, Regent Instrument, Inc., Québec, Canada). After that, the scanned root samples were oven-dried (at 60 °C) to determine constant weight (nearest 0.0001 g). Specific root length (SRL) was calculated as the total length divided by the corresponding dry mass. Root tissue density (RTD) was calculated as the dry mass divided by the corresponding total volume [47].

For anatomy, 10–15 leaves and 30 root tips in each FAA subsample were selected carefully for free-hand sectioning. All leaf and root cross-sections were observed using a compound microscope (ECLIPSE Ni-U Nikon, Tokyo, Japan) equipped with a C-SHG1 high-intensity mercury source (365 nm, Nikon, Tokyo, Japan), and only those with complete structures and distinct tissues were selected for subsequent analyses. For each leaf and root cross-section, traits (including leaf thickness, palisade thickness, spongy tissue thickness, vascular bundle thickness, the number and diameter of conduits in the main vein, the root diameter, cortex thickness, stele diameter, and the number and diameter of conduits in the stele) were measured to the nearest 1 μm using Motic Images Advanced 3.2 software. Specifically, the maximum conduit diameter was calculated as the average value of the first three largest conduits per leaf vascular bundle and root stele, respectively.

### 4.4. Data Analysis

For each species, the mean and standard error were calculated for leaf and root morphology and anatomy, respectively. As limited by the sample size of the species, a *t*-test (*p* = 0.05) was used to identify differences in these traits between woody and herbaceous species. At the same time, the significant differences (*p* < 0.05) among different species within the woody or herbaceous groups were identified according to Fisher’s LSD test. Correlations between pairs of functional traits across woody and herbaceous species were determined separately using the linear regression coefficient. To determine major axes of variation across multiple traits and identify whether there were concerted trait syndromes for woody or/and herbaceous species, we conducted a PCA on the trait data. All statistical analyses were performed using SPSS 19.0 (IBM Corp., Armonk, NY, USA), and data visualizations were created with SigmaPlot 10.0 (Systat Software Inc., San Jose, CA, USA) and ggplot2 [48].

## 5. Conclusions

Our study, which examined 18 common woody and herbaceous halophytes, revealed significant differences in both the trait variations and correlations of leaf and root morphology and anatomy between the two plant groups. Generally, similar to other species in a territorial ecosystem, woody species exhibited thicker root diameter, cortex thickness, and stele diameter, but lower specific root length and leaf area than herbaceous species in the Yellow River Delta. Both the cortex and stele were closely associated with root diameter, while the palisade tissue showed a positive correlation with leaf thickness. However, leaf and root traits appeared to function independently in the saline-alkali area. Further studies on trait variation and coordination are necessary, incorporating a broader range of species in saline environments, to enhance our understanding of plant adaptation to changing environmental conditions, and to supply a reference for species collections in vegetation restoration.

## Figures and Tables

**Table 1:** Descriptive statistics for root and leaf morphological and anatomical traits in 18 plant species in the Yellow River Delta in this study.

	Woody Species				Herbaceous Species				*p* Value
	Minimum	Maximum	Mean	CV	Minimum	Maximum	Mean	CV	
RD	0.12	0.44	0.30	0.34	0.13	0.40	0.22	0.36	0.050
SRL	33.25	526.47	220.97	0.82	88.69	1172.04	473.24	0.81	0.025
RTD	0.15	0.31	0.24	0.27	0.07	0.16	0.11	0.37	0.001
LT	150.15	675.87	286.99	0.75	107.63	1250.46	372.55	1.05	0.326
SLA	101.43	203.86	151.10	0.25	134.70	390.06	252.35	0.35	0.018
RCT	35.38	180.83	103.44	0.46	37.90	147.40	77.14	0.42	0.054
RST	40.82	111.20	76.75	0.31	28.46	89.85	50.78	0.39	0.005
RNC	4.44	30.33	9.22	0.89	4.22	16.27	8.41	0.52	0.366
RMC	3.21	7.33	5.89	0.26	2.49	9.30	5.25	0.41	0.238
SD	5.41	78.32	29.31	0.74	3.76	36.82	20.28	0.52	0.139
GCL	20.34	38.24	24.89	0.22	13.92	35.70	23.65	0.35	0.302
VD	1.37	21.91	12.46	0.68	0.90	1.71	1.25	0.22	0.001
LPT	57.29	94.22	72.52	0.20	22.89	205.10	93.03	0.98	0.265
LST	24.81	75.19	45.24	0.44	23.65	94.92	56.09	0.46	0.359
LVT	37.05	747.95	224.80	1.01	41.41	246.67	162.50	0.67	0.032
LMC	11.37	21.69	18.09	0.38	5.98	29.42	18.50	0.58	0.469

Note: RD, root tip diameter; SRL, specific root length; RTD, root tissue density; LT, leaf thickness; SLA, specific leaf area; RCT, root cortical thickness; RSD, root stele diameter; RNC, number of conduits in root; RMC, maximum diameter of root conduit; SD, stomatal density; GCL, guard cell length; VD, vein density; LPT, leaf palisade tissue thickness; LST, leaf spongy tissue thickness; LVT, leaf vascular bundle thickness; LMC, maximum diameter of leaf conduit; all these trait abbreviations are provided in Table 3. *p* values represent the comparison of means between woody and herbaceous species according to the *t*-test.

**Table 2:** Taxonomic list, life form, and vegetation types of 9 woody and 9 herbaceous species in the Yellow River Delta.

Species	Abbreviation	Life Form	Family
*Juniperus chinensis* L.	Juch	evergreen tree	Cupressaceae
*Platycladus orientalis* (L.) Franco	Plor	evergreen tree	Cupressaceae
*Melia azedarach* L.	Meaz	deciduous tree	Meliaceae
*Platanus* × *acerifolia* (Aiton) Willd.	Plac	deciduous tree	Platanaceae
*Pyrus betulifolia* Bunge	Pybe	deciduous tree	Rosaceae
*Styphnolobium japonicum* (L.) Schott	Stja	deciduous tree or shrub	Leguminosae
*Tamarix austromongolica* Nakai	Taau	deciduous tree or shrub	Tamaricaceae
*Tamarix chinensis* Lour.	Tach	deciduous tree or shrub	Tamaricaceae
*Apocynum venetum* L.	Apve	perennial erect subshrub	Apocynaceae
*Inula britannica* L.	Inbr	perennial herb	Asteraceae
*Lactuca tatarica* (L.) C. A. Mey.	Lata	perennial herb	Asteraceae
*Limonium sinense* (Girard) Kuntze	Lisi	perennial herb	Plumbaginaceae
*Cynanchum rostellatum* (Turcz.) Liede & Khanum	Cyro	perennial vine herb	Apocynaceae
*Glycine soja* Siebold & Zucc.	Glso	annual vine herb	Leguminosae
*Atriplex patens* (Litv.) Iljin	Atpa	annual herb	Amaranthaceae
*Echinochloa crus-galli* (L.) P. Beauv.	Eccr	annual herb	Poaceae
*Euphorbia humifusa* Willd.	Euhu	annual herb	Euphorbiaceae
*Suaeda salsa* (L.) Pall.	Susa	annual herb	Amaranthaceae

**Table 3:** Abbreviations and descriptions of examined root and leaf morphology and anatomical traits in this study.

Functional Trait	Abbreviation	Units	Description
Morphology			
Root tip diameter	RD	mm	Average diameter of root tip
Specific root length	SRL	m/g	Length per unit dry mass of root tip
Root tissue density	RTD	g/cm^3^	Mass per unit root volume of root tip
Leaf thickness	LT	μm	Average thickness of leaf
Specific leaf area	SLA	cm^2^/g	Area per unit dry mass of leaf
Anatomy			
Root cortical thickness	RCT	μm	Average thickness of cortex containing exodermis, endodermis, and cortical parenchyma cells
Root stele diameter	RST	μm	Average diameter of vascular cylinder
Number of conduits in root	RNC	No.	Number of conduits in the stele
Maximum diameter of root conduit	RMC	μm	Mean value of first three largest conduits per root stele
Stomatal density	SD	No./mm^2^	Total number of stomata per leaf area
Guard cell length	GCL	μm	Average length of guard cell in lower leaf epidermis
Vein density	VD	mm/mm^2^	Total vein length number of stomata per leaf area
Palisade tissue thickness	LPT	μm	Average thickness of palisade tissue in leaf cross-section
Spongy tissue thickness	LST	μm	Average thickness of spongy tissue in leaf cross-section
Leaf vascular tissue thickness	LVT	μm	Average thickness of leaf vascular bundle along the main vein
Maximum diameter of leaf conduit	LMC	μm	Mean value of first three largest conduits per leaf vascular bundle

## Data Availability

The data presented in this study are available on request from the corresponding author. The data are not publicly available due to privacy restrictions.
